# Diagnosing Breast Cancer with Microwave Technology: Remaining Challenges and Potential Solutions with Machine Learning

**DOI:** 10.3390/diagnostics8020036

**Published:** 2018-05-19

**Authors:** Bárbara L. Oliveira, Daniela Godinho, Martin O’Halloran, Martin Glavin, Edward Jones, Raquel C. Conceição

**Affiliations:** 1 Electrical and Electronic Engineering, National University of Ireland Galway, H91 TK33 Galway, Ireland; martin.glavin@nuigalway.ie (M.G.); edward.jones@nuigalway.ie (E.J.); 2 Instituto de Biofísica e Engenharia Biomédica, Faculdade de Ciências da Universidade de Lisboa, Campo Grande, 1749-016 Lisboa, Portugal; dgodinho94@gmail.com (D.G.); raquelcruzconceicao@gmail.com (R.C.C.); 3 Translational Medical Device Lab, National University of Ireland Galway, H91 TK33 Galway, Ireland; martin.ohalloran@nuigalway.ie

**Keywords:** machine learning, automated breast diagnosis, microwave imaging

## Abstract

Currently, breast cancer often requires invasive biopsies for diagnosis, motivating researchers to design and develop non-invasive and automated diagnosis systems. Recent microwave breast imaging studies have shown how backscattered signals carry relevant information about the shape of a tumour, and tumour shape is often used with current imaging modalities to assess malignancy. This paper presents a comprehensive analysis of microwave breast diagnosis systems which use machine learning to learn characteristics of benign and malignant tumours. The state-of-the-art, the main challenges still to overcome and potential solutions are outlined. Specifically, this work investigates the benefit of signal pre-processing on diagnostic performance, and proposes a new set of extracted features that capture the tumour shape information embedded in a signal. This work also investigates if a relationship exists between the antenna topology in a microwave system and diagnostic performance. Finally, a careful machine learning validation methodology is implemented to guarantee the robustness of the results and the accuracy of performance evaluation.

## 1. Introduction

### 1.1. Motivation

Microwave Breast Imaging (MBI) for breast cancer detection has seen significant academic and commercial development in recent years. At least 4 studies have reported findings from clinical trials [1,2,3,4,5,6,7], indicating that MBI has the potential to match state-of-the-art breast imaging methods, such as mammography. To date, the main goal of microwave imaging and signal processing algorithms has been the detection of tumours, i.e., to identify the presence of tumours within the breast, as shown by the literature in the area [8,9,10,11,12].

The development of automated breast diagnosis systems is relevant to the clinical environment, particularly considering recent reports showing minimal benefit of continuous mammographic screening in terms of long-term survival rates [13,14]. Many automated breast diagnosis systems have been proposed, and usually integrate signal or image pre-processing and segmentation, and diagnosis through machine learning [15,16]. Such systems have proved useful in aiding clinicians diagnose breast cancer, as they can identify features in a signal or image that may otherwise be missed through visual inspection. Automated diagnosis systems for microwave breast systems could play a key role in further establishing MBI as an early-stage breast cancer screening and monitoring method.

In the context of microwave breast diagnosis, a number of possibilities theoretically allow diagnosing breast tumours as benign or malignant. For example, the presence of microcalcifications in areas of the breast representing malignancy [17,18,19,20] and the difference in the dielectric properties between benign and malignant breast tumours [2,21]; however, further investigations characterising microcalcifications, and benign and malignant tissues in the microwave range are needed before microwave diagnosis systems based solely on these properties are viable. Finally, the shape and spiculation of tumours are widely recognised markers for their malignancy [22,23,24,25].

Benign tumours are roughly elliptical and usually have well circumscribed margins, and malignant tumours have irregular shapes and are surrounded by a radiating pattern of spikes, commonly referred to as spicules [22,23,24,25,26]. Previous studies have already shown how microwave backscattered signals may change if tumours of different sizes or shapes are present within the breast [27,28,29,30,31,32,33,34,35,36,37,38]. These studies have also demonstrated that classification and machine learning algorithms are able to learn from the shape differences in backscattered signals, albeit in relatively simple datasets. It is yet to be determined whether the performance of classification algorithms is adequate in clinically-complex scenarios.

This paper presents a comprehensive analysis into the fundamentals of microwave breast cancer diagnosis—as opposed to detection—systems. The main challenges are addressed, such as those arising from complex backscattered signals and appropriate machine learning methodology, and potential solutions are identified to overcome them. This work investigates, for the first time, whether a relationship exists between the predictive power of backscattered signals and the distribution of antennas in a microwave scan.

In the remainder of Section 1, the findings from previous studies using machine learning with microwave technology are reviewed in Section 1.2, and the main challenges still to be addressed in diagnosing breast tumours with microwaves are discussed in Section 1.3. The methodology is discussed in Section 2, which proposes a three-stage diagnosis system for addressing some of the primary challenges. The results are listed in Section 3 and Section 4 discusses and concludes the study.

### 1.2. Machine Learning and Microwave Technology: State-of-the-Art

With microwave breast prototype systems, a patient may sit or lie down while the breast is illuminated with low energy microwaves, and the resultant backscattered signals are recorded. In principle, it is possible to diagnose the type of tumour (benign or malignant) by examining the backscattered signals and recovering the tumour signature therein contained; in fact, previous studies indicate that backscattered signals may change if tumours of different sizes or shapes are present within the breast. In this section, a review is presented of the most significant studies to date to propose the use of machine learning to diagnose breast tumours based on their signatures.

In [29,30,31,32], several feature extraction methods (principal component analysis, discrete wavelet transforms, and independent component analysis) were used in combination with different classifiers (linear discriminant analysis, quadratic discriminant analysis, and supoprt vector machines) to diagnose breast tumours with backscattered signals. The analysis was based on numerical breast models composed mostly of adipose tissue; tumours were modelled with several sizes and shapes, and were located in the centre of the breast. These studies showed promise in using backscattered signals to diagnose tumours, and suggested that classifying tumour size ahead of tumour shape may improve diagnostic performance.

The suitability of neural networks to classify backscattered signals was also assessed. A combination of genetic algorithms and neural networks with discrete wavelet transforms was proposed in [33,34], and tested on a similar numerical dataset to the study above. As before, diagnostic performance was improved by separating tumours based on their size ahead of classification, and by investigating which transmit-receive antenna pairs provide the most useful information.

The same numerical dataset was also used in [35] to investigate the potential of self organising maps to track the development of a tumour from a benign state to different levels of malignancy. This study showed promise in distinguishing between different shapes of tumours.

In 2015, the authors of the present paper investigated the effect of signal pre-processing on diagnostic performance, by windowing the backscattered signal to contain only the tumour signature [38]. Clinically-realistic breast models were derived from the University of Wisconsin Computational Electromagnetics (UWCEM) repository [39], and tumour models of several sizes and shapes were located in various positions within the breast. The classification framework relied on principal component analysis in combination with support vector machines. The authors noted that the windowing methodology helped improve diagnostic performance when examining more complex and heterogeneous breast models.

Experimental datasets have also been used to assess the performance of diagnosis systems, namely by using principal component analysis in combination with support vector machines, linear discriminant analysis and quadratic discriminant analysis. In [36,37], tumour phantoms with various sizes and shapes were immersed in a breast phantom with dielectric properties matching those of adipose tissue. Importantly, the experimental results presented in these studies are in general agreement with previous numerical data.

The breast tumour diagnosis studies summarised in this section indicate that the shape of a breast tumour influences its signature within a backscattered signal, potentially allowing machine learning models to learn how to distinguish between benign and malignant tumours. These studies have also looked at the effect of intelligently using the most informative transmit-receive antenna pairs. In addition, these studies have concluded that it is beneficial to separate tumours according to size before final diagnosis, and also, that further signal pre-processing methodologies should be explored when dealing with more complex breast models, for example, breast models with increased content of glandular tissue.

Additionally, other authors have implemented comparable machine-learning approaches for detection, i.e., to determine whether a tumour is present in the breast [40,41,42,43,44,45,46,47,48,49]. While an in-depth review of the detection studies based on machine learning performed to date is beyond the scope of this work, their main findings are summarised here for completeness. Detection studies indicate that there is sufficient information in the backscattered signals to inform about the presence of a tumour. These studies show that detection performance can be improved by using differential signals which highlight the tumour signature and by extracting time-frequency features of the signals ahead of classification. Similarly to the diagnosis studies discussed above, selecting the transmit-receive antenna pairs with the most meaningful classification information also seems to positively impact detection performance.

### 1.3. Challenges with Microwave Breast Diagnosis Systems

In the previous section, a review was presented of the main microwave studies that use machine learning to diagnose breast tumours as benign or malignant. In this section, the remaining challenges in the development of microwave breast diagnosis systems are discussed, as well as potential solutions, from two perspectives: addressing the complexity of backscattered signals gathered in clinically-realistic conditions; and developing a validation methodology for the classification models.

#### 1.3.1. Complexity of Clinically-Realistic Data

Benign and malignant tumours may present a wide range of sizes, shapes and spiculations at their margin, which can change the backscattered energy received at a given antenna. In addition, the shape of the human breast changes from person to person, and so does the distribution of adipose and glandular tissues inside the breast, which effectively alters the attenuation along each propagation path. This diversity leads to equally diverse backscattered signals, making the design of a single platform for diagnosis a complex task. Some of the challenges related to breast and tumour composition can be summarised as follows:

(i) Difficulty in capturing the tumour signature from the backscattered signal due to: (1) presence of skin, the response of which can be orders of magnitude larger than the tumour signature; (2) presence of glandular tissue clusters, which can be confused with tumour tissue, due to similarities in composition (water content and generally higher dielectric properties); (3) tumours can occur in different locations within the breast, embedded in various breast structures.
(ii) Differences in the tumour signature for a given transmit-receive antenna pair due to: (1) tumours of different shapes, meaning antennas in different locations have a different view of the tumour; (2) various angles between transmit and receive antennas, which can affect the phase of the tumour signature; (3) varying distances between the antennas and the edge of the tumour.

Particularly regarding Section 1.3.1, a number of strategies have already been proposed in previous studies. Artefact removal algorithms have been proposed, which deal with large skin reflections and decrease the glandular tissue influence on the backscattered signals, for example [50,51]. As mentioned in Section 1.2, previous studies have also suggested that: pre-processing signals by means of windowing could highlight and time-align the tumour signature [38]; extracting features based on time-frequency representations of the data could further capture the essence of the tumour signature while disregarding the background noise [48,49]; and classifying a dataset according to tumour size before attempting at classification based on the level of malignancy [29,30,31,33].

Concerning Section 1.3.1, while some studies have observed an improvement in diagnostic performance by restricting the classification to the backscattered signals captured with the most informative transmit-receive antenna pairs [33,41,43,45,46,47], no thorough investigation of optimal antenna topology and optimal use of the information from each channel was found in the literature.

A further set of challenges exists in translating microwave breast diagnosis systems to experimental and clinical evaluation: patient positioning and movement; intra-patient variation due to menstrual cycle and hormonal changes; inter-patient variation in breast size, shape and composition.

#### 1.3.2. Challenges in Building Robust Machine Learning Classification Models

Ideally, a machine learning algorithm trained with a particular dataset should be generalisable to new, unseen datasets. Common practice is that a model should first be trained on a subset of the data, and then tested on another unseen subset of the data. The training set should be as large as possible, to minimise the variance in training the model, but the unseen subset of the data should also be representative of the original dataset, so the performance evaluation is meaningful.

However, performance evaluation commonly observed in the literature is prone to variations in approach, and often some degree of error, leading to overly-optimistic performance reports. Poor model validation is often due to: (1) overfitting of the learning model during the training phase; (2) overfitting during model selection; and (3) contamination of the information across the dataset.

Cross-validation has long been regarded as a good method to prevent overfitting of the model during training, and it is widely used as the basis for model selection. However, it has also been shown that using the performance obtained from cross-validation during model selection as the overall performance of the model might be overly-optimistic, and not generalisable. This effect is often referred to as selection bias [52,53].

Careful construction of a machine learning-based system should also consider the type of pre-processing and feature-based algorithms applied to the original dataset. As noted in the previous section, the extraction of features from the original dataset could be key to diagnostic performance; however, pre-processing or feature-based methods could also play a part in the contamination of information between the training and test sets. Typically, to prevent contamination, any method involving computation of the relationship across multiple observations, should first be applied to the train set, and the training transformations should then be applied onto the test set.

Many of the issues listed above have not been explicitly addressed in previous studies proposing detection or diagnosis algorithms through machine learning for microwave breast systems. Implementing careful and consistent methodologies for model validation and performance evaluation should, however, become best practice. Ultimately, creating learning models without proper validation methodologies could compromise the usability of microwave breast diagnosis systems.

## 2. Materials and Methods

**Figure 1:**
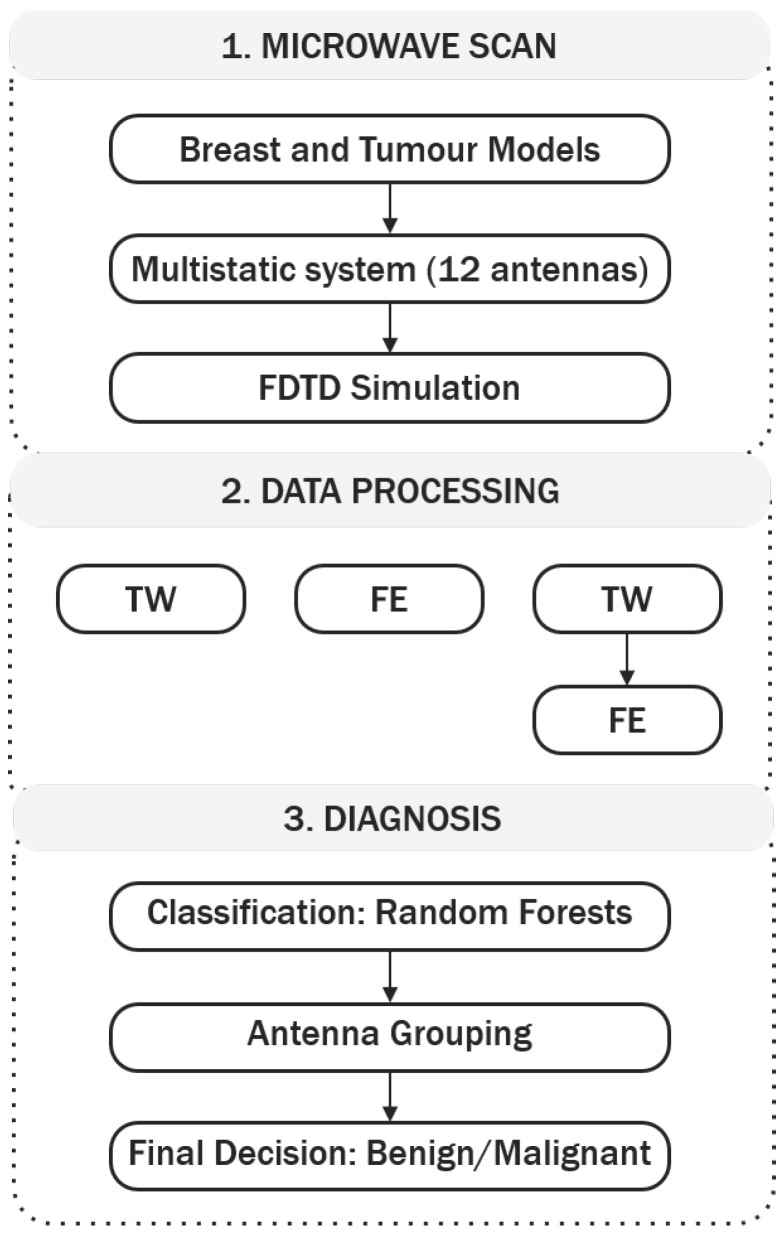
3-stage diagnosis platform implemented in this study. Stage 1 consists of data collection in a microwave breast prototype. Stage 2 consists of data processing by means of tumour windowing (TW) and feature extraction (FE); the relative importance of each algorithm is compared by applying TW in combination with FE, or TW only, or FE only. Stage 3 is the diagnosis stage, which uses random forests as the classifier, includes an antenna grouping algorithm, and ends with a final diagnosis of benign or malignant.

In this study, the authors have implemented an integrated methodology of detecting and diagnosing breast tumours using backscattered signals. The proposed methodology is 3-fold, comprising data acquisition, data processing and diagnosis. The overall diagnostic architecture is depicted in Figure 1.

Stage 1 consists of the microwave breast scan. To address some of the issues in dealing with clinically-realistic datasets (as highlighted in Section 1.3.1), a data processing stage was implemented next (Stage 2), comprising a tumour windowing approach to select signal segments of interest, combined with feature extraction. The relative benefits of both algorithms are analysed by comparing the diagnostic performance of applying one of the following: only tumour windowing; only feature extraction; tumour windowing in combination with feature extraction, i.e., feature extraction performed after the tumour signature is windowed from the original backscattered signal.

Stage 3 consists of the diagnosis and encompasses classification of the dataset through a range of techniques, including random forests, antenna grouping, and final decision as benign or malignant. The authors explore the concept introduced in previous studies that some channels (i.e., transmit-receive antenna pairs) might be more useful to improve diagnostic performance, by implementing three classification models, where each classification model makes different use of the information from each channel. The three classification model types will be described in greater detail in Section 2.3.2. With the algorithms implemented in Stage 3, this study aims to understand: (1) if the angular distance between the transmit and receive antennas in a channel determines its predictive power; (2) if the distance between the tumour and the channel has an impact on diagnostic performance; (3) finally, how to better use the information from each channel while adhering to best machine learning practices. In addition, a careful model validation methodology was implemented in Stage 3, to prevent issues like the ones detailed in Section 1.3.2. The three stages of the proposed microwave breast diagnosis platform will be described in greater detail in the following sub-sections.

### 2.1. Microwave Scan—Breast and Tumour Modelling and Electromagnetic Simulation

For the purposes of this study, a numerical dataset of breast and tumour models was created through electromagnetic simulation with the Finite-Difference Time-Domain (FDTD) formulation. This method is well-established in the literature and widely used in the field of microwave breast cancer imaging to model the propagation and scattering of microwave signals within the breast [54].

MRI-derived breast models were taken from the repository created by the UWCEM laboratory [39]. All breast models in the repository are mapped to the dielectric properties of normal and malignant breast tissues as established by Lazebnik et al. [55]. In total, 3 heterogeneous breast models were used in this study. In terms of percentage composition, the breast models used in this study range between 1% to 27% of glandular tissue by volume of breast, with the remainder percentage of tissue corresponding to adipose tissue.

For the creation of tumour models, the clinically-informed tumour modelling algorithm previously developed by the authors [56] was used to generate 72 unique tumour models, with average sizes ranging from 6 mm to 20 mm in diameter. Several degrees of spiculation were used to create tumours grouped into two distinct classes: smooth borders to represent benign tumours (with 0≤s≤0.25), and spiculated borders for malignant tumours (with 0.50≤s≤0.90), where *s* is the spiculation parameter from [56] with 0≤s≤1. The tumours were placed in 5 different positions within the breast as described in medical reports, corresponding to locations in the four breast quadrants and the central portion.

The electromagnetic measurement system was modelled with a concentric ring of equally-distanced 12 Hertzian dipole antennas around the breast in a fully multistatic setup (which means the angle between two adjacent antennas is 30°. Each antenna element is modelled as an electric current source. The antennas were immersed in a medium with dielectric properties equivalent to those of adipose tissue. The FDTD simulations were performed using a differentiated Gaussian pulse with centre frequency of 6 GHz and a −3 dB) bandwidth (of 6 GHz). The spatial resolution of the system is 1 mm, and the sampling frequency is 600 GHz. Additionally, a reference simulation was also performed. This reference signal is later used to remove antenna effects in the backscattered signals from simulations of the full breast with tumours.

**Figure 2:**
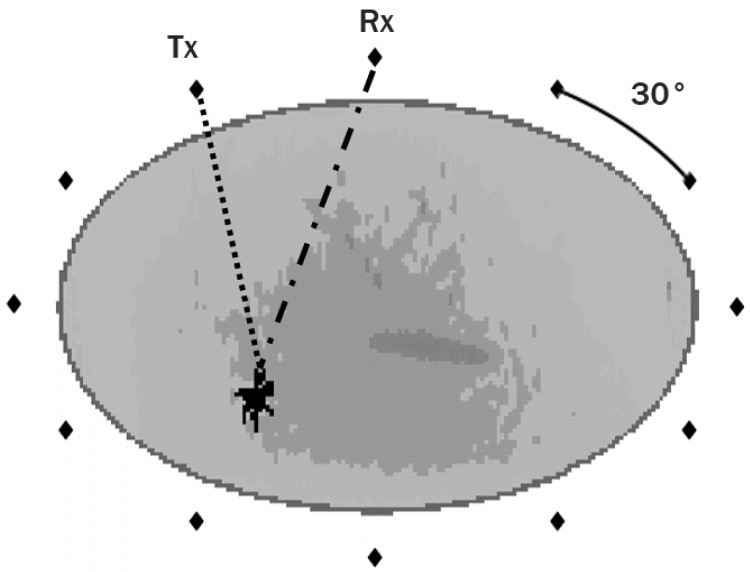
Representation of the acquisition setup designed for this study, where the antennas are represented by the black diamonds surrounding the breast. A coronal slice of one of the breast models is shown; the breast has fibroglandular tissue in the interior, and a malignant tumour in one of the lower breast quadrants (represented by the black spiculated shape). The path from one transmitting antenna (Tx) to the tumour and from the tumour to a receiving antenna (Rx) is shown in the dash and dot-dash lines, respectively; the 30° angle between two adjacent antennas is also shown.

Figure 2 displays a schematic representation of the acquisition setup designed for FDTD simulation in this study, where the antennas are represented by the black diamonds surrounding the breast. A coronal slice of one of the breast models used in the study is shown, including fibroglandular tissue in the interior, and a malignant tumour in one of the lower quadrants (the spiculated shape in black). To aid the visualisation of the setup, the path from one transmitting antenna (Tx) to the tumour and from the tumour to the receiving antenna (Rx) is shown in dash and dot-dash lines, respectively.

With the proposed setup, one microwave breast scan is composed of backscattered signals collected from 78 independent channels. In total, 1080 microwave scans were performed (3 breast models each combined with 72 tumour models in 5 different positions within the breast). A dataset containing a total of 84,240 backscattered signals is used in this study.

### 2.2. Data Processing

This section describes the processing methods used to prepare the data ahead of classification. Two methods are used to process the backscattered signals, either by means of tumour windowing or feature extraction.

#### 2.2.1. Tumour Windowing

In this paper, the authors expand on the tumour windowing concept initially proposed in [38]. Once the tumour location is identified, the round-trip propagation delay between the tumour and each channel is calculated, based on the average propagation speed through three media: immersion medium, skin and interior of the breast; the estimated tumour response is then windowed from the backscattered signal. In this paper, the ideal tumour location is used. The approximate window length was decided empirically. Visual assessment of a subgroup of backscattered signals gathered with different tumour models embedded in breast models with varying background contents found that a window length of 2.5 times the pulse width is appropriate to extract the full tumour response from the signals.

The propagation delay is highly dependent on the average dielectric properties of each medium; consequently, reflections yielding from different tumours propagating through different paths will be hard to align. To compensate for this effect, the windowing algorithm looks for the peak energy in each backscattered signal, and time-aligns the tumour responses on this basis. Each windowed tumour response is finally downsampled to a sampling frequency of 30 GHz. After downsampling, the window length of the tumour signatures consisted of 60 time samples.

By implementing the proposed tumour windowing algorithm: the reflection from the skin is eliminated; a high level of clutter resulting from the glandular clusters is potentially removed; signals collected from different channels are time-aligned. As a result, the tumour response is isolated, potentially simplifying the task given to the classification algorithm. To compensate for antenna effects in the signals, an artefact removal step can be performed prior to windowing.

When only tumour windowing is applied during Stage 2 of the 3-stage diagnosis platform (Figure 1, TW), the windowed time-domain signatures are treated as independent observations, which are then passed as input to the classification model.

#### 2.2.2. Feature Extraction

Feature extraction is frequently applied to capture meaningful information embedded in a signal, and is helpful in reducing the dimensionality of the problem when compared to the original data.

Visual analysis of backscattered signals reveals that benign tumours result in signals that tend to preserve the original morphology of the gaussian peak, while malignant tumours result in more irregular signals, due to increased reflections from tumour spicules. Therefore, this paper examines the use of a set of features that capture signal morphology and frequency content for diagnosis. The proposed feature extraction method relies on peak analysis of different time and frequency representations of the original data, where each group of features is calculated for the signal collected by each channel of each scan. As the extraction of features is done independently on each observation, no calculations are made across the dataset and between tumour signatures, which prevents accidental data contamination issues, as those described in Section 1.3.2.

**Figure 3:**
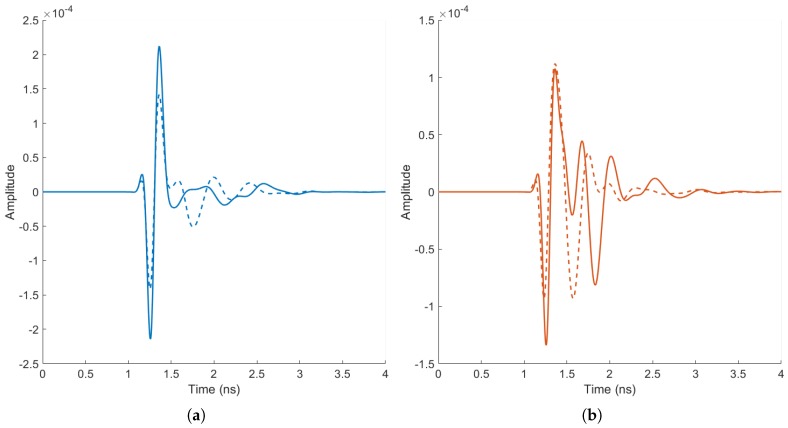
Example of tumour signatures from benign tumour models (**a**) and malignant tumour models (**b**), captured in ideal conditions, with a fully adipose breast model. In (**a**), the benign tumour signatures are smooth, and the shape of the gaussian curve is preserved to a reasonable extent; In (**b**), the malignant tumour signatures are subject to a greater degree of irregularity, exhibiting an increased number of peaks.

By way of example, Figure 3 displays some of the differences identified by visual analysis of benign (Figure 3a) and malignant signatures (Figure 3b). The signals were collected under ideal conditions to highlight the potential differences between types of tumours, with an adipose-only breast model; for both tumour types, two tumour models were simulated of different sizes and shapes. The resultant signals have been time-aligned and windowed. As observed in Figure 3a, the backscattered signals from the benign tumour models exhibit little distortion and the original Gaussian shape is preserved well; conversely, in Figure 3b, the malignant tumour models result in backscattered signals with a higher level of waveform distortion.

In total, 30 features were extracted from each signal, divided into four sub-groups, as shown in Table 1. If feature extraction is performed on the original backscattered signals, the method is referred to as FE; if feature extraction is performed after the backscattered signals have been processed with the tumour windowing algorithm, the method is referred to as TW + FE.

### 2.3. Computer Aided Diagnosis

This section describes, in detail, Stage 3 (Diagnosis) of the 3-stage microwave diagnosis platform described in Figure 1. An overview of random forests, the classification algorithm, is first provided in Section 2.3.1. Section 2.3.2 describes the three types of classification models implemented in this study. The antenna grouping algorithm is detailed in Section 2.3.3. The validation methodology is described in Section 2.3.4, and the metrics to assess diagnostic performance are discussed in Section 2.3.5.

#### 2.3.1. Classification Algorithm: Random Forests

In this study, random forests [62] were implemented to classify backscattered signals as benign or malignant.

The method of random forests is an ensemble method that essentially works by generating many single classification trees [63] and outputting the class that is the mode of the classes of all individual trees. Each tree is grown (i.e., trained) using a randomly sampled subset of observations and features from the entire dataset. Due to the inherent randomness in the process, the generated trees are uncorrelated, which ultimately contributes to the algorithm’s low bias and low variance. Random forests provide generalisable models that tend not to overfit, are quick to run and are easy to interpret [62].

For the operation of a random forest, one-third of the observations in the original dataset are left out when training each tree. These observations are referred to as out-of-bag (oob) and are used as a separate set to assess the performance error of each tree. The out-of-bag error provides a measurement of the generalisation ability of the process, which is useful, for example, when optimising the internal parameters of the random forest. Random forests also allow measuring the importance of each feature in the training of each tree. In the context of diagnosing backscattered signals as benign or malignant, a measure of feature importance could provide the means to further refine classification models.

In this study, the following hyperparameters of the random forest were optimised to ensure good trained models: number of trees, number of features, leaf size. A Bayesian optimisation algorithm was implemented to perform the search for the best hyperparameters. The best hyperparameters were deemed to be those yielding the smallest out-of-bag misclassification error (that is, the hyperparameters yielding the highest accuracy).

#### 2.3.2. Antenna Topology: Types of Classification Models

Although all channels in a given scan may contain information about a tumour, the tumour signature varies between channels depending on: the location of the tumour relative to the antennas in a channel; and the relative distance between the transmit and receive antennas in a channel. The angular distance between transmit and receive antennas in a channel is referred to as channel angle in the remainder of this work.

**Figure 4:**
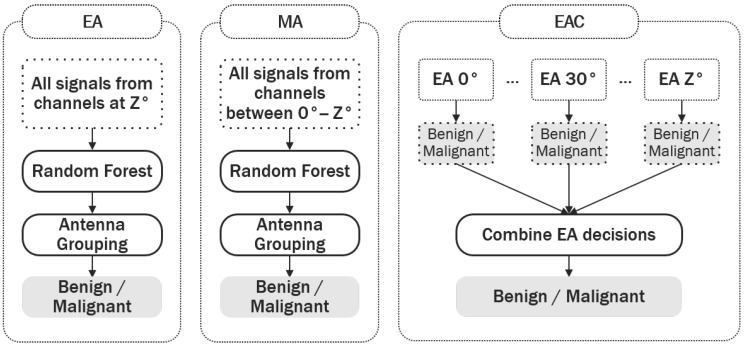
Description of the three types of classification models implemented: EA (equal angles), MA (multiple angles) and EAC (equal angles combined). The classification models vary in the way signals from different channel angles are utilised by the classification algorithm, where Z represents the channel angle (Z varies between 0° and 180°, and increases in steps of 30°). EA models only classify signals from a single channel angle. MA models classify signals collected at channels where the transmit and receive antennas are separated by different angles. Through majority voting, EAC models combine the predictions from multiple EA models at different channel angles to produce a final diagnosis.

This variance in the tumour signatures between channels may impact the performance of the classification model, as the variance between channels may be as large as the variance between the signatures of benign and malignant tumours. To explore the significance of intra-channel variance, three types of classification models were designed, which differ in the way signals from different channel angles are utilised by the classification algorithm. The three types of classification models are shown in Figure 4. Differences in the performance of the three types of classification models may help identify if an optimal antenna pair topology exists in terms of the channel angle, which can ultimately contribute to improving diagnostic performance.

The assumed system architecture is as described in Section 2.1, with one ring of 12 antennas equally distributed around the breast. Let *Z* be the angle between the transmit and receive antennas in a channel; here, Z∈[0,180]°, and *Z* increases by steps of 30°.

Equal Angle (EA) classification models only receive information from channels with an equal angle between transmit and receive antennas. Seven EA models were built to assess if channels with equal angles contribute to a higher diagnostic performance.

Multiple Angle (MA) models use information from channels where transmit and receive antennas are separated by different angles. If such a model underperforms, it will serve as an indication that the information captured by channels with different angles varies significantly, and that the classification model cannot adequately learn the similarities within benign and malignant tumours across signals collected at different angles. In total, six MA models were built using all antenna pairs in the interval [0,i.Z]°, where Z=30° and i=1,2,…,6, until all antenna pairs were used.

Equal Angle Combined (EAC) models use all possible EA models (one for each channel angle), and the predictions from each one are combined (through majority voting) at the end to produce a final diagnosis. By combining the predictions from each individual model, models which yield an incorrect result are likely to be disregarded, ultimately contributing to an increase in diagnostic performance. As before, a total of six EAC models were built using all antenna pairs in the interval [0,i.Z]°, with Z=30°, until all antenna pairs were used. Table 2 summarises the models of each type, in particular the range of angles considered in this study.

#### 2.3.3. Antenna Grouping

At this stage, it is important to define how backscattered signals (or the features extracted from each backscattered signal) are used in the decision-making process.

Each patient scan is comprised of signatures collected from 78 different channels (as per the system architecture described in Section 2.1), which are classified independently. However, in a realistic, clinical diagnostic system, a diagnosis is given based on a full scan, and not on the basis of a single signature. This means that the independent channel predictions need to be combined to form a final diagnosis. In the existing literature, either the procedure in determining the final diagnosis is not thoroughly discussed, or the diagnostic performance is reported based on the results from the independent channels.

To address this, an antenna grouping algorithm is implemented in this study, by which the predictions of the independent channels are grouped, and a majority vote is completed to determine if a scan is benign or malignant. The advantages of implementing such an algorithm are two-fold.

Firstly, with microwave diagnosis systems, the possibility should be considered that a signal comprises lower quality information about the tumour shape, which could result in incorrect predictions about its malignancy (e.g., signals from channels that may have poor signal-to-noise ratios). By implementing the antenna grouping algorithm, a mechanism is created that effectively allows disregarding incorrect predictions from lower quality channels.

Secondly, channels closer to a tumour should intuitively produce more useful information for its diagnosis. By implementing a ranked version of the antenna grouping algorithm, it is possible to investigate if the proximity between tumour and channel translates into higher diagnostic performances. The ranked version of the antenna grouping algorithm operates as follows. Let *W* be the number of channels used to perform antenna grouping, ordered by proximity to the tumour. Antenna grouping is performed by increasing *W* in steps of 1, until all available channels are used. For example, if W=3, the majority vote is taken from the signals collected by the 3 channels closest to the tumour, before concluding on the final diagnosis.

#### 2.3.4. Assessing Diagnostic Performance

**Figure 5:**
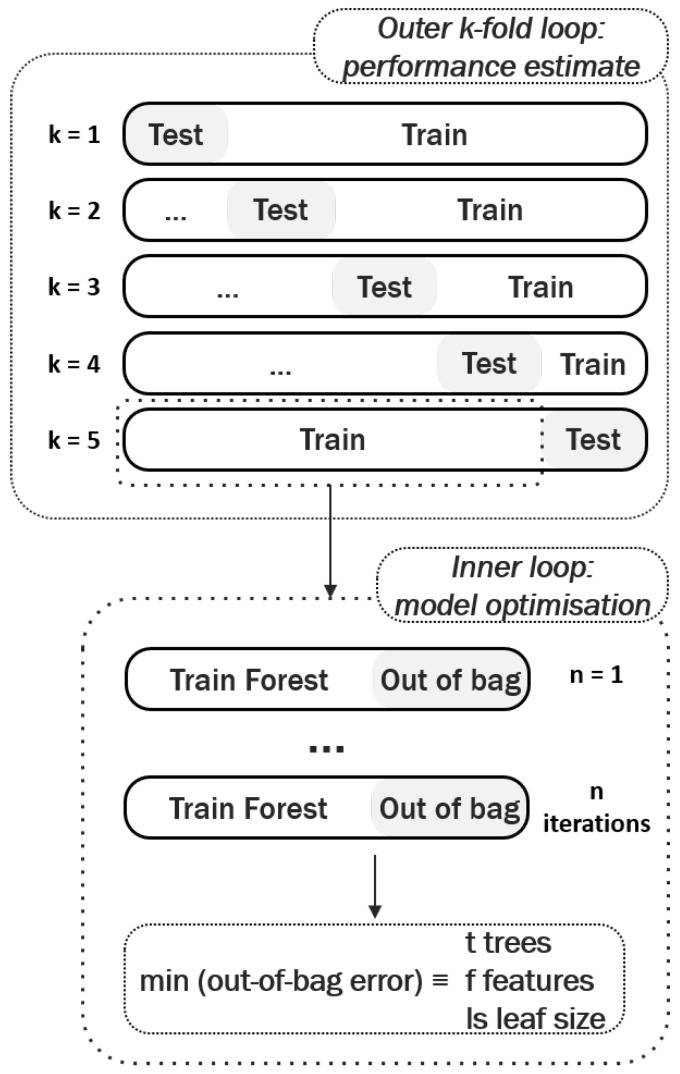
Nested cross-validation methodology implemented in this study to perform model optimisation and estimate model performance. In each fold, the random forest model is optimised on the train set, and new predictions are made on the test set. The predictive power of the model corresponds to the average performance obtained in the test sets across all outer folds.

In this study, the authors have implemented a validation methodology based on the idea of nested cross-validation [53] to assess diagnostic performance, and mitigate sources of contamination when optimising the classification model. It has been shown that nested cross-validation helps prevent overly optimistic reports of model performance [52,53]. An overview of the process is shown in Figure 5, and can be summarised as follows:

- The entire dataset is divided into *k* stratified folds, containing equal representations of each class. In this study, the authors chose k=5 outer folds as it offers a good compromise between a statistical performance analysis and speed of implementation. All signals from one breast scan are kept together when splitting each fold into training and test.
- For each outer fold, the model is trained and the classifier hyperparameters optimised. As previously detailed, random forests directly provide the out-of-bag error, which serves as an unbiased estimate of the model performance when optimising its hyperparameters. When using other classifiers another inner cross-validation loop can be implemented at this stage.
- The predictive power of the model is then reported as the average performance obtained in the test sets across all outer folds.

#### 2.3.5. Performance Metrics

In this study, the performance of a classification model is assessed by plotting the Receiver Operating Characteristic (ROC) curves. ROC curves are created by plotting the false positive rate achieved by the classification model in the horizontal axis, against the true positive rate in the vertical axis [64]. ROC curves provide with a simple graphical representation of the diagnostic ability of the classification model, by varying the decision threshold that is used in producing the final binary decision, i.e., whether breast tumours are benign or malignant.

The Area Under the ROC Curve (AUC) is also used as a measure of classification performance. Generally, the higher the AUC, the more generalisable the model is, and the better it performs.

## 3. Results

This section is divided into three sub-sections. Section 3.1 discusses the issue of antenna topology and antenna grouping. Here, a relationship between the channel angle (angle between transmit and receive antennas) and predictive power is investigated, resulting in the proposal of a useful method to use the information from several multistatic scan channels. In Section 3.2, the effect of increasing tissue heterogeneity on overall diagnostic performance is discussed. Section 3.3 identifies possible avenues to expand on the knowledge gained with the extraction of features.

### 3.1. Antenna Topology

This section details the analysis of optimal antenna topology to be used in a breast model containing 5% of glandular tissue by volume.

**Figure 6:**
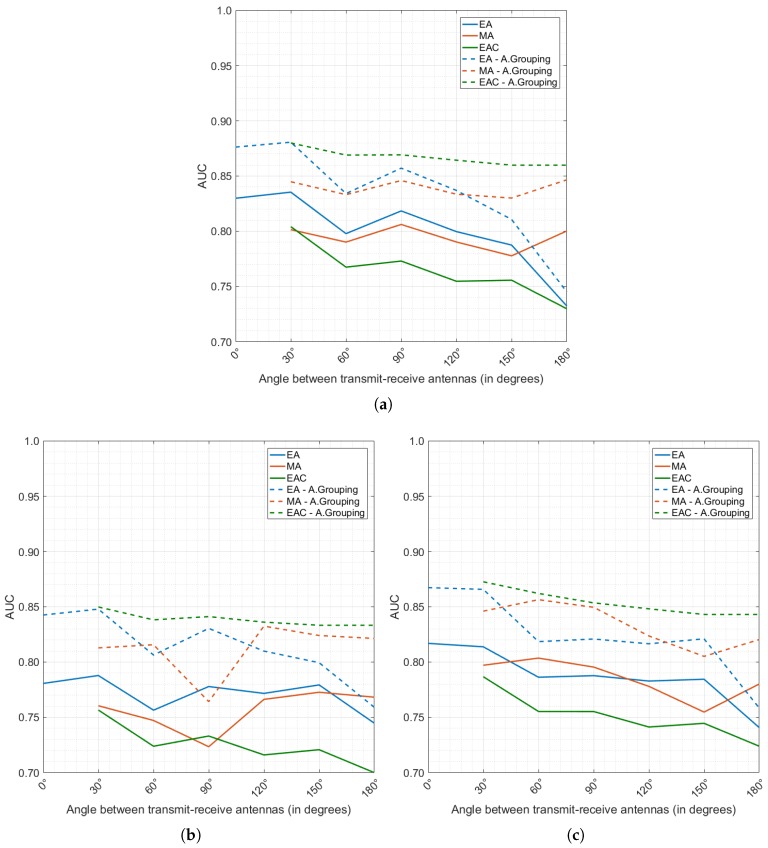
Diagnostic performance for the EA (blue), MA (orange) and EAC (green) models produced when features are extracted from the original dataset: (**a**) TW; (**b**) TW + FE; (**c**) FE. A.Grouping refers to the antenna grouping algorithm (using all available channels towards the majority vote). The full lines correspond to the diagnostic performance when antenna grouping was not applied, and the dashed lines when antenna grouping was applied. The horizontal axis shows the channel angles used to build each model; for the MA and EAC models, the models contain all channel angles between 0° and the angle shown in the horizontal axis.

Three types of classification models were defined in Section 2.3.2: EA, MA, EAC. Figure 6a–c detail the diagnostic performance achieved by all models produced, for each of the processing methods under analysis, TW, TW + FE and FE, respectively. The effect of antenna grouping (as defined in Section 2.3.3) is also investigated in Figure 6, by comparing diagnostic performance before antenna grouping (full lines) and after the antenna grouping algorithm is applied (dashed lines), using all available channels in the majority vote.

Firstly, the positive impact of antenna grouping is clearly noticeable. The diagnostic performance when antenna grouping is applied is always superior (as shown by the dashed lines in Figure 6). By taking the majority vote of all individual decisions from one single breast scan, a minority of incorrect predictions are cancelled by a majority of correct classifications. A more in-depth analysis of the effect of the ranked version of the antenna grouping algorithm (not shown in Figure 6 for conciseness) reveals that at least 3 channels are necessary to achieve a reliable diagnostic performance; above 3 channels, the performance tends to stabilise and only minor improvements are observed at the cost of more complex models. This result is seen across all classification model types (EA, MA and EAC), and by applying either of the pre-processing methods (TW, TW + FE and FE).

In Figure 6, it is also noticeable that EA and EAC models generally seem to outperform MA models. This result confirms the hypothesis that classification models perform better when dealing with signals collected under the same conditions:

- With the TW pre-processing method (Figure 6a), tumour windowing and time alignment of the signals have been performed; however, it is likely that the TW processing is not sufficient to completely neutralise the inherent differences from channels at different angles, especially considering that the intra-channel variability is likely to increase when noisy experimental or clinical data is used. One additional factor to consider with the TW processing is that knowledge of tumour location is fundamental, and localisation errors might also impact accurate time-alignment of tumour signals from different channel angles;
- In the TW + FE and FE pre-processed datasets (Figure 6b,c, respectively), comparable conclusions are observed. Models classifying signals from the same channel angles perform better. In addition, the dataset pre-processed only with FE, which does not require previous knowledge of the tumour location, slightly outperforms the TW + FE pre-processed dataset.

It is also interesting to note that channel angles below 90° in the EA models lead to higher diagnostic performance when compared to channels at higher angles, which might indicate that reflected backscattered signals keep more information about tumour shape than transmitted signals. EAC models seem to benefit from this; when combining information from individual EA models, the predictions made by the EA models at lower channel angles dominate, ultimately contributing to disregarding incorrect predictions made at higher channel angles. Regardless of the pre-processing method, the best result seems to be achieved with EAC 0–30°.

In summary, optimal diagnostic performance is achieved when EAC models were used, particularly when combining channels with reflected backscattered signals. Antenna grouping is needed to achieve one final diagnosis per scan, and it helps increase diagnostic performance of the system, as it provides the means to disregard random incorrect predictions. Using all channels in the antenna grouping algorithm provides with best performance, although, the authors observed that most of the relevant information is contained in the channels closest to the tumours.

### 3.2. Effect of Tissue Heterogeneity

Increasing tissue heterogeneity is a concern when designing platforms for the diagnosis of breast cancer based on microwave backscattered signals. As glandular and tumour tissues are both characterised by higher dielectric properties, the response due to glandular clusters in the breast might sometimes be confused with the response of a tumour, causing an increased rate of false positives. In this study, the authors examine if the proposed windowing and time-alignment methodology is sufficient to handle breast heterogeneity, and if the extraction of the above-mentioned features provides meaningful and sufficient information.

**Figure 7:**
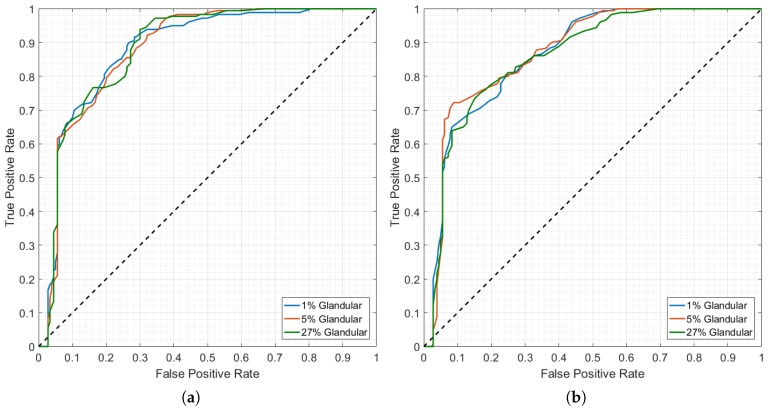
ROC curves showing diagnostic performance with the EAC 0–30° classification model using all channels in antenna grouping, for (**a**) TW dataset; (**b**) FE dataset. The blue line corresponds to the performance of a breast model with 1% glandular tissue by volume, orange line 5%, and green line 27%. The black dotted line represents the null hypothesis in the ROC curve.

From Section 3.1, one of the best performing antenna topologies was that in the EAC model at 0–30°, using all available channels when performing antenna grouping; here, the TW and FE processing methods performed the best among all considered tests. The effect of increasing tissue heterogeneity is shown in Figure 7, by plotting ROC curves obtained for the TW dataset (Figure 7a), and FE dataset (Figure 7b) using the optimal antenna topology. Separate classification models were built to diagnose scans from breast models with 1% (blue line), 5% (orange line) and 27% (green line) of glandular tissue by volume.

The random forest classifier appears to be robust to tissue heterogeneity. The average performance across breast models with increasing glandular tissue content is comparable, when using either the TW or the FE methods during the pre-processing stage of the system. However, in an experimental or clinical setup, the performance of the TW pre-processed dataset is likely to decrease as tissue heterogeneity increases; noisier experimental backgrounds lead to an increased number of reflections, and the localisation of the tumour signature in the backscattered signal might be affected. Conversely, the extracted features are able to capture the differences between benign and malignant tumours, even with signals recorded in more heterogeneous breast models.

Finally, the ROC curves indicate that diagnostic performance may also be optimised by varying the decision threshold. Commonly used as a fixed threshold of 0.5, the range of optimal decision thresholds identified in this study range between 0.36 and 0.52 (not shown in Figure 7 for conciseness), which could carry further importance when translating the microwave diagnosis system to clinic.

### 3.3. Relative Feature Contribution

Previous sections examined the effect of antenna grouping, and the impact of tissue heterogeneity on the best performing system from initial baseline tests. In this section, an analysis of feature selection is presented, by means of the relative feature contribution map provided as one of the outputs of the random forest classifier. Investigating which features mostly contribute to the training of each tree inside a random forest could help refine the classification models, ensuring their robust and stable performance in complex scenarios, such as in experimental systems prone to high noise levels.

**Figure 8:**
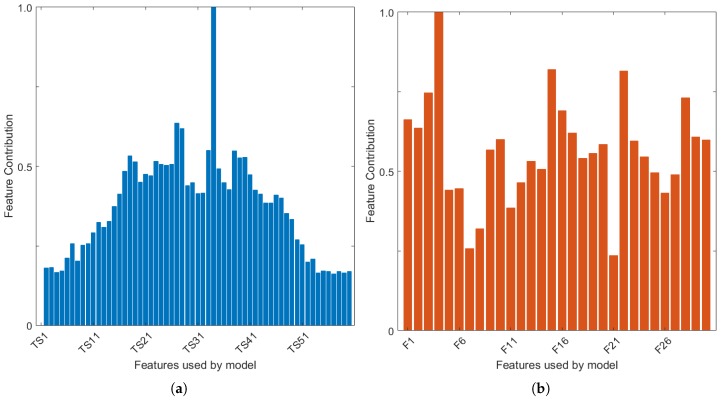
Map of relative feature contribution calculated during the training of the random forest model for the breast model with 27% glandular content by volume. The EAC 0–30°, with all channels in antenna grouping, is used. (**a**) refers to the dataset pre-processed with TW method; the horizontal axis shows the time samples (TS) which make up the time-domain tumour responses; (**b**) refers to the dataset pre-processed with the FE method, where F1 to F30 shown in the horizontal axis correspond to Features 1 through 30.

In Figure 8a,b, the relative feature contribution map is shown, for the breast model with 27% glandular tissue, for the TW and FE pre-processed datasets respectively. Classification was performed with the EAC 0–30° model, which uses all channels in the antenna grouping algorithm. This model is shown as an example, although the authors observed similar feature contributions across all breast and classification models.

Firstly, Figure 8a (TW) shows that the classification model using tumour windowing is heavily reliant on one single feature. This feature is time sample 34 in the example shown. All classification models used in this study display the same reliance on one feature, which varies between time sample 33 and 36. This result suggests that any errors in the tumour windowing and alignment algorithm could indeed have a large impact in the performance of the classification.

Regarding classification with the FE method (Figure 8b), a larger number of features appear to contribute to the performance of the random forest model. Feature #4 (location of the negative peaks in the tumour signature) ranks highest, which is visible across all classification and breast models used in this study. Nine other extracted features are also identified as being particularly important in the training of the classification models. In decreasing order of contribution: amplitude of the negative peaks in the tumour signature (feature 3 shown in Figure 8b), amplitude and mean full-width half-maximum (FWHM) of positive peaks (#1, #11), mean amplitude of the peaks in the auto-correlation curve (#22), integral of the tumour signature (#16), integral of the absolute value of the signature (#17), mean power spectral density using the Welch estimate (#25), mean amplitude and FWHM of the peaks in the periodogram (#28, #29).

The features listed above were some of the features to mostly contribute to the classification of shape and spiculation of benign and malignant tumours. Particularly, the authors note the contribution of the features derived from the autocorrelation and power spectral density analysis, which reflect information otherwise not available in the time-domain tumour signatures.

## 4. Discussion and Conclusions

Microwave breast diagnosis systems could play a key role in further establishing microwave breast imaging and diagnosis as a tool for continuous and safe breast cancer monitoring. While diagnosis of breast tumours as benign or malignant could theoretically be performed through a number of avenues, shape and spiculation at the margin of a tumour are widely accepted as markers for malignancy, and previous studies have already demonstrated how backscattered signals are influenced by the shape of a tumour.

In this study, the authors extend previous analysis to further confirm that diagnosis of microwave backscattered signals by means of machine learning is feasible, however, there are many factors that affect performance and system optimisation must be performed with care.

Firstly, antenna grouping was identified as a key step for an increased diagnostic performance. Individual signals which compose one breast scan are independent observations, and are classified accordingly receiving a label of benign or malignant. By performing antenna grouping, those individual predictions are grouped into one final diagnosis (majority vote) for each scan. The results of this study show the benefit behind this approach, as, by doing so, a mechanism is created to disregard minor incorrect predictions. In addition, the authors observed that a relatively small number of antennas closest to the tumour guarantee the correct prediction; above this number of channels, performance tends to stabilise, but importantly, does not decrease. In this study, the 3 channels closest to the tumour were found to yield correct diagnosis; however, this may change in microwave prototypes or equipment with different setups.

Secondly, the results showed how signals collected at channels with different angles between the transmit and receive antennas have to be appropriately used by the classification model. By building individual classification models that only classify signals from channels with the same angle, diagnostic performance is increased. The predictions of individual classification models can later be combined into a fused-type model, which once again contributes to increasing the diagnostic performance. In addition, the results also showed how channels containing reflected backscattered signals performed better over channels with transmitted signals. In this study, the optimal channel angle was 0–30°.

Thirdly, data pre-processing was also shown to have an impact on diagnostic performance. When dealing with time-domain signals, knowledge of tumour location is required. With this information, a tumour windowing and time-alignment algorithm can be implemented to isolate the tumour response, while decreasing the influence of the background. A new set of 30 features was also proposed, which are extracted per backscattered signal; these features mostly rely on peak analysis of the time-domain signal, and of the frequency content of the signal. Both methods performed comparably, however, in practice, additional factors come into play which will impact performance:

- With currently available algorithms, localisation of the tumour signature in the response could be prone to error, which would impact the performance of the tumour windowing and could ultimately decrease diagnostic performance. This factor should not be neglected when building systems to classify time-domain signals.
- The extraction of features performed well, even when the time-domain signal was not pre-processed by windowing. This method appears as an alternative when exact tumour location is not available to the user. In addition, a reduced set of features of maximised contribution was identified, which could lead the way into finding an optimal set of features towards more robust classification models for microwave systems.

Finally, good machine learning practice is extremely important when designing microwave breast diagnosis systems. Without adequate feature processing methods and model validation strategies, reports of performance could be overly optimistic and not reproducible, ultimately impeding clinical acceptance of microwave diagnosis tools.

Further investigations are needed to assess the robustness of microwave breast diagnosis systems given the complexities of experimental and clinical data, such as, patient positioning and movement, intrapatient variation due to menstrual cycle and hormonal changes, and interpatient variation in breast size, shape and composition.

## Figures and Tables

**Table 1:** Description of all 30 features used in this study, divided into four sub-groups: Time-domain features, Autocorrelaton features, Power spectral density (PSD) features based on Welch’s method, and PSD features using the periodogram method.

Time-Domain Features, Calculated From the Windowed Signals
#1–#4 Amplitudes and locations of the maximum positive and negative peaks
#5 Variance
#6 Root-mean-squared error
#7–#8 Number of positive and negative peaks
#9–#10 Mean amplitude of the positive and negative peaks
#11–#12 Mean full-width half-maximum (FWHM) of the positive and negative peaks
#13–#14 Mean separation between positive and negative peaks
#15 Number of zero crossings
#16 Integral of the signal
#17 Integral of the absolute value of the signal
#18 Positive percentage area of the signal
#19 Negative percentage area of the signal
**Autocorrelation features**, which involves calculating the autocorrelation sequence of each signal [57,58]. The following features are then extracted from the autocorrelation sequence
#20 Mean value of the autocorrelation sequence
#21 Number of peaks in the autocorrelation sequence
#22 Mean amplitude of the peaks
#23 Mean FWHM of the peaks
#24 Mean separation between the peaks
**PSD features**—estimate of the psd of the signal, using Welch’s method [59]
#25 Mean value of the Welch estimate
**PSD features**—estimate of the psd of the signal, using the periodogram method [60,61]
#26 Mean value of the periodogram estimate
#27 Number of peaks in the periodogram
#28 Mean amplitude of the peaks
#29 Mean FWHM of the peaks
#30 Mean separation between the peaks

**Table 2:** Summary of the total of number of classification models built for this study, and the channel angles used in each model. In EA models, only signals from channels at the specified angle are used in the process. In MA models, all signals from channels in the specified range are used. In EAC models, individual EA models in the specified range are combined through majority voting to produce a final diagnosis.

Classification Model Number	EA	MA	EAC
(1)	0°	-	-
(2)	30°	0–30°	0–30°
(3)	60°	0–60°	0–60°
(4)	90°	0–90°	0–90°
(5)	120°	0–120°	0–120°
(6)	150°	0–150°	0–150°
(7)	180°	0–180°	0–180°

## Abbreviations

AUC	Area Under receiver operating characteristic Curve
EA	Equal Angle
EAC	Equal Angle Combined
FDTD	Finite-Difference Time-Domain
FE	Feature Extraction
FWHM	Full-Width Half-Maximum
MA	Multiple Angle
MBI	Microwave Breast Imaging
PSD	Power Spectral Density
ROC	Receiver Operating Characteristic
TW	Tumour Windowing
UWCEM	University of Wisconsin Computational Electromagnetics
